# Prominent and conspicuous astrocyte atrophy in human sporadic and familial Alzheimer’s disease

**DOI:** 10.1007/s00429-023-02707-x

**Published:** 2023-09-20

**Authors:** J. J. Rodríguez, F. Zallo, E. Gardenal, Joan Cabot, X. Busquets

**Affiliations:** 1 Functional Neuroanatomy Group; IKERBASQUE, Basque Foundation for Science, Department of Neurosciences, Medical Faculty, University of the Basque Country (UPV/EHU), 48009/48940 Bilbao/Leioa, Vizcaya Spain; 2 Laboratory of Molecular Cell Biomedicine, Department of Biology, University of the Balearic Islands, 07122 Palma, Spain

**Keywords:** Alzheimer’s disease, Sporadic Alzheimer’s, Familial Alzheimer’s, Entorhinal Cortex, Astrocytes, Atrophy, GFAP, GS

## Abstract

Pathophysiology of sporadic Alzheimer’s disease (SAD) and familial Alzheimer’s disease (FAD) remains poorly known, including the exact role of neuroglia and specifically astroglia, in part because studies of astrocytes in human Alzheimer’s disease (AD) brain samples are scarce. As far as we know, this is the first study of a 3-D immunohistochemical and microstructural analysis of glial fibrillary acidic protein (GFAP)- and glutamine synthetase (GS)-positive astrocytes performed in the entorhinal cortex (EC) of human SAD and FAD samples. In this study, we report prominent atrophic changes in GFAP and GS astrocytes in the EC of both SAD and FAD characterised by a decrease in area and volume when compared with non-demented control samples (ND). Furthermore, we did not find neither astrocytic loss nor astrocyte proliferation or hypertrophy (gliosis). In contrast with the astrogliosis classically accepted hypothesis, our results show a highly marked astrocyte atrophy that could have a major relevance in AD pathological processes being fundamental and key for AD mnesic and cognitive alterations equivalent in both SAD and FAD.

## Introduction

The pathophysiology of Alzheimer’s disease (AD) in both the sporadic (SAD) and familial (FAD) forms still remain ill-known. Indeed, genetics and a variety of environmental influences play a role in AD aetiology and cerebral amyloid angiopathy (Braak and Braak 1991; Rodríguez et al. 2016). FAD (5% AD) is characterised by mutations in the amyloid precursor protein (APP), presenilin 1 (PSN1) and presenilin 2 (PSN2) (Barber 2012), whereas SAD (95% of AD) is associated to mutations in thousands of genes (Barber 2012). On the other hand, astrocyte and microglial activation are also related with the pathogenesis of AD. In fact, astrogliosis is classically considered as the astrocytic reaction to AD pathology in human brain and animal models (Rodríguez et al. 2016; Nagele et al. 2003).

The distinctive cellular events in SAD and FAD are amyloid beta peptide (Aβ) accumulation, Tau neurofibrillary tangles and their hyperphosphorylation, which triggers the so-called neurotoxic cascade (Karran et al. 2011) neuroinflammation and neuronal death (Selkoe and Hardy 2016). On the other hand, extracellular Aβ induces astrogliosis, and microstructural analysis shows that astrocytes are found in the core of the amyloid plaque and its surroundings, affecting the physiology of astrocytes and impairing cognition (Rodríguez et al. 2016).

However, our group has shown that, in the triple transgenic mouse model of AD (3xTg-AD), a generalised astroglia atrophy exists with just a restricted astrogliosis in the vicinity of Aβ plaques, in addition to glutamate glial metabolism and trophic factors alterations in the hippocampus (HPC), but not in the entorhinal cortex (EC) and prefrontal cortex (PFC) showing a differential region behaviour that also implies the involvement of Glutamate transporter and receptors (Rodríguez et al. 2016).

In this context, the glutamate balance via the glutamate–glutamine (Glu–Gln) shuttle is critical for cognitive functions and excitotoxicity in AD being glutamine synthetase (GS) a key element in the Glu–Gln cycle without forgetting the (Glu–Gln-GABA metabolism cycle) fundamental for both excitatory and inhibitory transmission (Eid et al. 2012). Our group has reported a reduction of GS-immunoreactive astrocytes in two major cognitive areas of the HPC (DG and CA1) in 3xTg-AD. This reduction of GS-immunoreactive astrocytes was paralleled by a decrease in global GS suggesting alteration of glutamate balance (Olabarría et al. 2011). On the contrary, we did not find changes in GS-immunoreactive astrocytes or double-labelled GS/GFAP in the same animal model in the EC (Yeh et al. 2013).

Despite the 3xTg-AD we used in these previous studies shows similar distribution of Aβ and Tau alterations present in human AD and demonstrates synaptic dysfunction with impaired long-term potentiation (LTP; Oddo et al. 2003), the interpretations of these studies are limited since these are not performed in human pathological samples. In this work, we carried out a 3-D anatomical, immunohistochemical and microstructural analysis of glial fibrillary acidic protein (GFAP)- and glutamine synthetase (GS)-positive astrocytes performed in the entorhinal cortex (EC) study using post-mortem human brain samples of patients who suffered from SAD and FAD compared with ND. Our results show the relevance of astrocyte atrophy in human AD which are equivalent to the observed in animal models, suggesting that in the human pathology, astrocyte atrophy is fundamental to the cognitive and mnesic alterations in early and late onset of AD contributing to neuronal damage and CNS dysfunction shown by cytoskeletal alterations—GFAP—and metabolic dysfunctions—GS—(Eid et al. 2012; Rodríguez et al. 2016, 2023). Therefore, we consider that development of therapeutic strategies treating both astrocytes and neurons would improve their altered connectivity, function together with synaptic functionality and interaction, including abnormal accumulation of Gln driving to potential astroglial toxicity through hyperammonemia (Eid et al. 2012). Thus, this innovative approach and potential treatment would hopefully reduce the deleterious effects of this devastating disease.

## Material and methods

### Human material

Post-mortem human AD brain samples ranging from 40 years of age till over 80 of both SAD (*n* = 8) and their equivalent non-demented controls (ND, *n* = 5) were obtained from the Netherlands Brain Bank, whereas FAD (*n* = 9) brain samples were obtained from the University of Antioquia, in Colombia (kindly provided by Profs. Francisco Lopera and Diego Sepulveda). All human samples were obtained from the Netherlands Brain Bank (NBB) and from the University of Antioquia, Colombia (UA), according to their guidelines and ethical committees’ approval. Briefly, all donors singed the Informed Consent form, gave permissions for post-mortem brain autopsies and for the use of their brain material and medical records for research purposes. The informed consent form of the NBB and UA meets all current legal and ethical requirements for brain autopsy, tissue storage and use of tissue and clinical data for scientific research worldwide (https://www.brainbank.nl/).

All samples were fixed by immersion in a solution of 4% paraformaldehyde (Sigma, Germany) and 0.1 M phosphate buffer (PB) pH 7.4. Then, they were cut into 40–50-μm-thick coronal sections using a vibrating microtome (MICROM HM 650 V, Thermo Scientific, USA). Free-floating brain sections in 0.1 mM PB, pH 7.4 were collected and stored in a cryoprotectant solution containing 25% sucrose and 3.5% glycerol in 0.05 M PB at pH 7.4.

### Double immunofluorescence for human paraffin-embedded sections

Sections were incubated in 30% methanol and 3% hydrogen peroxide in 0.1 M PB at pH 7.4 for endogenous peroxidase inactivation, following by incubation in 0.3 M glycine in 0.1 M PB for autofluorescence elimination. Once rinsed with 0.1 M PB, tissue slides were treated with 1% sodium borohydride in 0.1 mM PB to remove the excess of aldehyde groups and subsequently, blocked in 0.5% bovine serum albumin in 0.1 M Tris Saline (TS) with 1% Triton-X pH 7.6. Then, they were incubated during 48 h at room temperature (RT) in the primary single and/or antibodies cocktail (Rabbit anti-GFAP, 1:10,000, Sigma and Mouse anti-GS, 1:2000, Millipore). After that, sections were incubated during 2 h at RT in the corresponding secondary antibodies (Goat anti-Rabbit AlexaFluor 594 and Goat anti-Mouse AlexaFluor 48, 1:400, Invitrogen). Finally, sections were rinsed with 0.1 mM PB and cover slipped using Vectashield.

### Tri-dimensional reconstruction of astrocytic morphology and population count

Single GFAP-, single GS- and GFAP/GS-immunopositive astrocytes of the entorhinal cortex were imaged using a confocal microscope (Leica TCS STED CW SP8 microscope), at least 35 per sample. Parallel confocal planes were superimposed and morphological analysis was carried out by Cell Analyst (Chvátal et al. 2007) using digital filters (average 3 × 3, convolution, gauss 5 × 5) to determine the surface area (S) and volume (V) of the GFAP, GS and GFAP/GS-stained astrocytes.

To determine whether the changes in the PFC GFAP-positive astrocytes, GS and GFAP/GS cytoskeletal surface area and volume are linked with changes in the number of astrocytes expressing these markers, we determined the numerical density (Nv, Number of cells/mm^3^) of astrocyte population in at least three representative non-consecutive sections, analysing an area of 600,000 μm^2^ in coronal sections of 40 μm thickness, thus representing a total volume of 24,000,000 μm^3^ per section (Olabarría et al. 2011; Yeh et al. 2013). GFAP-, GS- and GFAP-positive astrocytes were intensively labelled against a dark background that made them easy to identify with an equal chance of being counted. The number of GFAP-, GS- and GFAP-positive astrocytes was determined and quantified blindly on fluorescent microscope images by a single observer to reduce counting bias to a minimum.

### Statistical analysis

One-way Analysis of Variance (ANOVA) with the post hoc Tukey test was performed to examine the significant alterations in the morphometric parameters of the different astroglial populations between groups. Data are expressed as mean ± SEM. Significance was accepted at *p* < 0.05. Initially, we also compared the potential statistical differences in the age difference onset of SAD patients 65–75 years and 75 years onwards). As there was no difference, we amalgamate all cases as a unique group.

## Results

### Changes in GFAP-positive astrocytes morphology in SAD and FAD compared to ND human brain samples

**Fig. 1:**
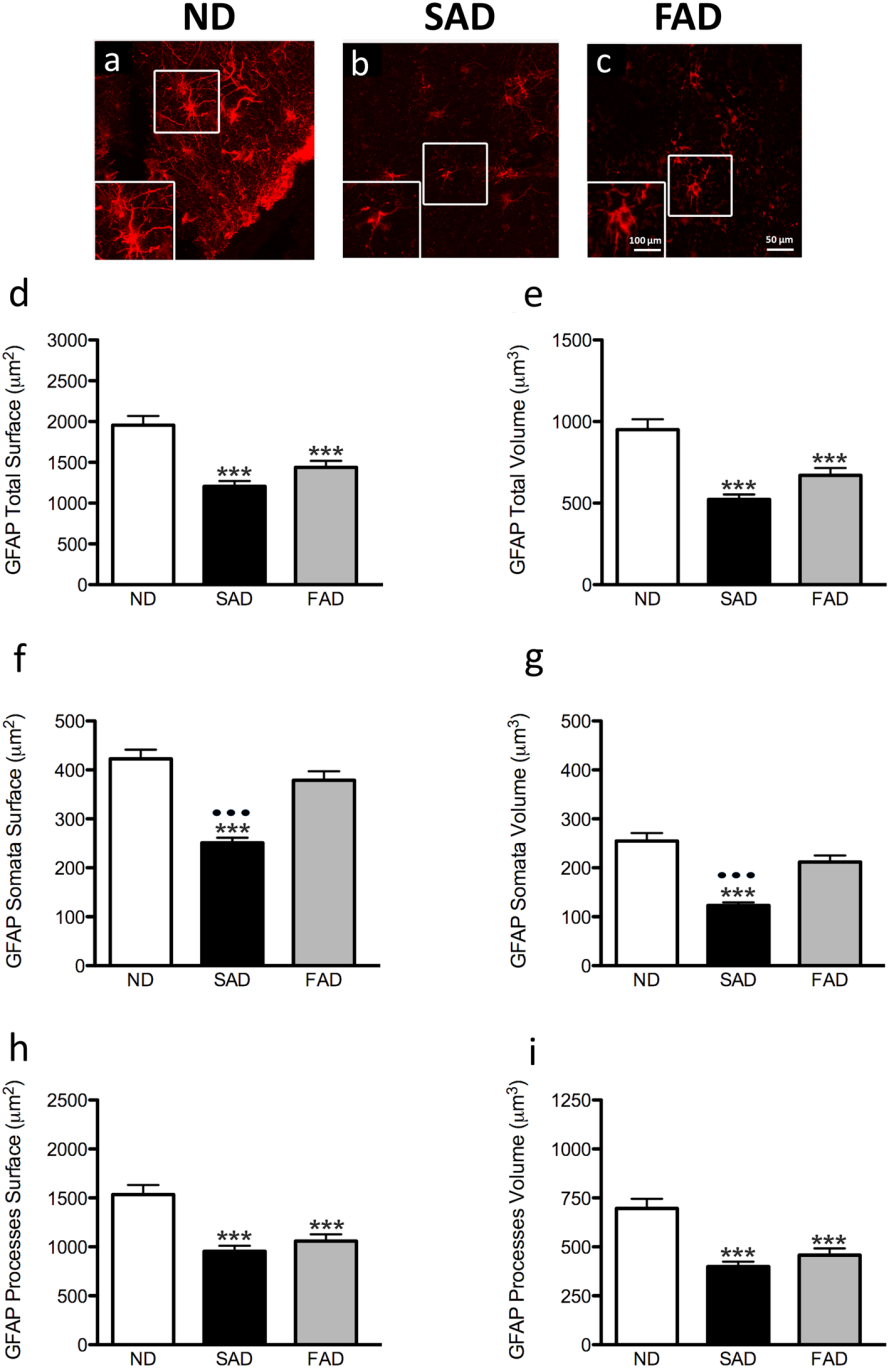
Confocal micrographs illustrating GFAP immunoreactive astrocytes cytoskeleton in the human entorhinal cortex of ND subjects (**a**), SAD (**b**) and FAD (**c**). Bar graphs showing the astrocytic cytoskeleton total surface (**d**), total volume (**e**), somata surface (**f**), somata volume (**g**), processes surface (**h**) and processes volume (**i**). Bars represent means ± SEM (***p ≤ 0.001, comparing SAD or FAD with ND and *p* ≤ 0.001, comparing SAD with FAD)

GFAP-positive astrocytes analysis of FAD and SAD human entorhinal cortex revealed differences of total surface area and volume, processes surface area and volume as well as somata surface and volume compared to control ND human EC slides [F_3,386_ = 14.23, 16.17, 27.51, 26.64, 11.55, 12.84, respectively; *p* < 0.001] (Fig. 1, Table 1). When compared ND to SAD, we found a decrease of 38.29% of the total GFAP surface area (1956.0 ± 111.5 µm^2^ *vs.* 1207 ± 63.43 µm^2^; *p* < 0.001; Fig. 1d). Similar decrease of surface area was observed when comparing ND with FAD samples (26.43%; 1956.0 ± 111.5 µm^2^ *vs.* 1439 ± 80.63 µm^2^; *p* < 0.001; Fig. 1d). Accordingly, the total volume of GFAP-positive astrocytes was significantly reduced when comparing ND to SAD with a 45% decrease (950.9 ± 62.94 µm^3^ *vs.* 523.0 ± 29.69 µm^3^; *p* = 0.001; Fig. 1e) or when comparing ND to FAD (29.53%; 950.9 ± 62.94 µm^3^ *vs.* 670.1 ± 45.01 µm^3^, *p* < 0.001; Fig. 1e). The somata surface also showed a significant 40.65% decrease when comparing ND to SAD (422.9 ± 18.48 µm^2^ *vs.* 251 ± 10.42 µm^2^; *p* < 0.001; Fig. 1f) or a non-significant change when comparing ND to FAD (422. 9 ± 18.48 µm^2^ *vs.* 379.1 ± 18,20 µm^2^; *p* > 0,05; Fig. 1f); despite the evident tendency to decrease as shown by a 10–15% reduction. On the other hand, somata volume showed a significant reduction of 51.63% when comparing ND to SAD (254.7 ± 16.56 µm^3^ *vs.* 123.2 ± 6,37 µm^3^; *p* < 0.001; Fig. 1g) and non-significant changes when comparing ND to FAD (254.7 ± 16.56 µm^3^ *vs.* 212,1 ± 13.18 µm^3^; *p* > 0.05; Fig. 1g) notwithstanding the evident tendency to decrease as shown by a 10–15% reduction. We also find an extremely interesting difference between SAD and FAD, since both somata surface and volume were also reduced in SAD compared to FAD (251 ± 10.42 µm^2^ *vs* 379.1 ± 18,20 µm^2^; *p* < 0,001; 123.2 ± 6,37 µm^3^ *vs* 212,1 ± 13.18 µm^3^; *p* < 0.001, respectively).

**Table 1:** Changes in GFAP-positive astrocytes morphological parameters in SAD and FAD compared to ND human brain samples

	ND vs SAD		ND vs FAD		SAD vs FAD	
Processes surface	1533 ± 98.07 vs 956.2 ± 56.10	*p* < 0.001	1533 ± 98.07 vs 1060 ± 67.60	*p* < 0.001	956.2 ± 56.10 vs 1060 ± 67.60	ns
Somata surface	422.9 ± 18.48 vs 251 ± 10.42	*p* < 0.001	422.9 ± 18.48 vs 379.1 ± 18.20	ns	251 ± 10.42 vs 379.1 ± 18.20	*p* < 0.001
Total surface	1956 ± 111.5 vs 1207 ± 63.43	*p* < 0.001	1956 ± 111.5 vs 1439 ± 80.63	*p* < 0.001	1207 ± 63.43 vs 1439 ± 80.63	ns
Processes volume	696.2 ± 49.17 vs 399.8 ± 24.90	*p* < 0.001	696.2 ± 49.17 vs 458.1 ± 33.88	*p* < 0.001	399.8 ± 24.90 vs 458.1 ± 33.88	ns
Somata volume	254.7 ± 16.56 vs 123.2 ± 6.37	*p* < 0.001	254.7 ± 16.56 vs 212.1 ± 13.18	ns	123.2 ± 6.37 vs 212.1 ± 13.18	*p* < 0.001
Total volume	950.9 ± 62.94 vs 523 ± 29.69	*p* < 0.001	950.9 ± 62.94 vs 670.1 ± 45.01	*p* < 0.01	523 ± 29.69 vs 670.1 ± 45.01	ns

Values represent mean ± SEM and *P*-value. Astrocytic processes, somata and total surface mean and SEM are expressed as µm^2^, whilst astrocytic processes, somata and total volume mean and SEM are expressed as µm^3^. ns = non-significance

A detailed analysis of the GFAP-positive astrocytic processes demonstrated a reduction of both surface and volume of these processes. When compared ND to SAD, we found a decrease of 37.63% of processes’ surface area (1533 ± 98.07 µm^2^ *vs.* 956.2 ± 56.10 µm^2^; *p* < 0.001; Fig. 1h). Similarly, a reduction was found in processes’ surface area when comparing NAD to FAD (30.86%; 1533 ± 98.07 µm^2^ *vs.* 1060.0 ± 67.60 µm^2^, *p* < 0.001; Fig. 1h). The volume of GFAP-positive processes was also reduced. When compared ND to SAD, we found a decrease of 42.56% of the total processes’ volume (696.2 ± 49.17 µm^3^ *vs.* 399.8 ± 24.90 µm;^3^ *p* < 0.001; Fig. 1i). Similar decrease of processes’ volume was also observed when comparing ND with FAD samples (34.2%; 696.2 ± 49.17 µm^3^ *vs.* 458.1 ± 33.88 µm^3^; *p* < 0.001; Fig. 1 i), whilst the changes were equivalent when compared both SAD and FAD (Fig. 1h and i).

### Changes in GS-positive astrocytes morphology in SAD and FAD compared to ND human brain samples

**Fig. 2:**
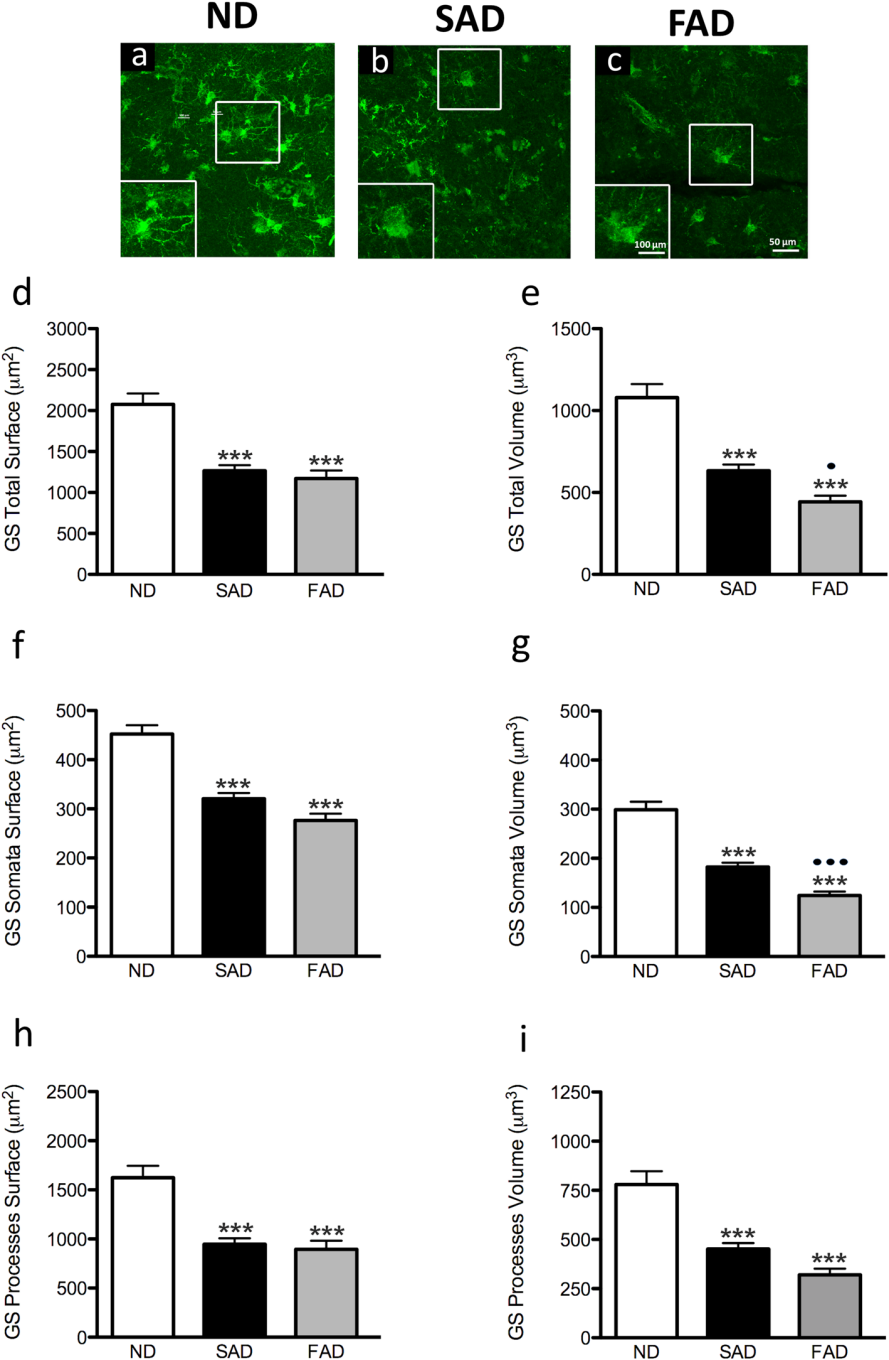
Confocal micrographs illustrating GS-immunoreactive astrocytes morphology in the human entorhinal cortex of ND subjects (**a**), SAD (**b**) and FAD (**c**). Bar graphs showing the astrocytic total surface. (**d**), total volume (**e**), somata surface (**f**), somata volume (g) processes surface (**h**) and processes volume (**i**) Bars represent means ± SEM (***p ≤ 0.001, comparing SAD or FAD with ND and ⋅*p* ≤ 0.05, *p* ≤ 0.001, comparing SAD with FAD)

GS-positive astrocytes analysis of FAD and SAD human EC samples revealed a loss of total surface area and volume as well as processes surface area and volume compared to control ND human EC slides [F_3,364_ = 28.22, 38.20, 29.05, 47.91, 26.01, 33.55, respectively; *p* < 0.001] (Fig. 2, Table 2). When compared ND to SAD, we found a decrease of 39.0% of the total surface area (2077.0 ± 132.1 µm^2^ *vs.* 1267.0 ± 66.97 µm^2^; *p* < 0.001; Fig. 2d). Similar decrease of surface area was observed when comparing ND with FAD samples (43.62%; 2077.0 ± 132.1 µm^2^ *vs.* 1171.0 ± 97.31 µm^2^ *p* < 0.001; Fig. 2d). Accordingly, the total volume of GS-positive astrocytes was significantly reduced when comparing ND to SAD with a 41.22% decrease (1078.0 ± 81.70 µm^3^ *vs.* 633.7 ± 36.51 µm^3^; *p* < 0.001; Fig. 2e) or when comparing ND to FAD (58.87%; 1078.0 ± 81.70 µm^3^ *vs.* 443.4 ± 36.61 µm^3^; *p* < 0.001; Fig. 2e). We also found differences between SAD and FAD total volume (633.7 ± 36.51 µm^3^ *vs* 443.4 ± 36.61 µm^3^; *p* < 0.05; Fig. 2e). The somata surface also showed a significant 29.15% decrease when comparing ND to SAD (452.5 ± 17.82 *vs.* 320.6 ± 11.66; *p* < 0.001; Fig. 2f) and a decrease of 38.92% when comparing ND to FAD (452.5 ± 17.82 µm^2^ *vs.* 276.4 ± 14 µm^2^; *p* < 0.001; Fig. 2f). On the other hand, somata volume showed a significant reduction of 39.08% when comparing ND to SAD (299.1 ± 15.94 µm^3^ *vs.* 182.2 ± 8.98 µm^3^; *p* < 0.001; Fig. 2g) and a decrease of a 58.48% when comparing ND to FAD (299.1 ± 15.94 µm^3^ *vs.* 124.2 ± 7.89 µm^3^; *p* < 0.001; Fig. 2g). When compared the total volume of GS astrocytes and their somata, we observe a further reduction in SAD when compared to FAD (182.2 ± 8.98 µm^3^ *vs* 124.2 ± 7.89 µm^3^; *p* < 0,001; Fig. 2 g). A detailed analysis of the GS-positive astrocytic processes demonstrated a reduction of both surface and volume of these processes. When compared ND to SAD, we found a decrease of 41.75% of processes’ surface area (1625.0 ± 119.6 µm^2^ *vs.* 946.6 ± 59.94 µm^2^; *p* < 0.001; Fig. 2h). Equally, we found a reduction in processes surface area when comparing SAD to FAD (44.94%; 1625.0 ± 119.6 µm^2^ *vs.* 894.7 ± 88.0 µm^2^; *p* < 0.001; Fig. 2h). As indicated, the volume of GS-positive processes was also reduced. When compared ND to SAD, we found a decrease of 42.06% of the total processes volume (779.2 ± 68.36 µm^2^ *vs.* 451.5 ± 29.85 µm^2^; *p* < 0.001; Figure 2i). Similar decrease of processes volume was observed when comparing ND with FAD samples (59.04%; 779.2 ± 68.36 µm^2^ *vs.* 319.2 ± 31.68 µm^2^; *p* = 0.001; Fig. 2i).

**Table 2:** Changes in GS-positive astrocytes morphological parameters in SAD and FAD compared to ND human brain samples

	ND vs SAD		ND vs FAD		SAD vs FAD	
Processes surface	1625 ± 119.6 vs 946.6 ± 59.94	*p* < 0.001	1625 ± 119.6 vs 894.7 ± 88	*p* < 0.001	946.6 ± 59.94 vs 894.7 ± 88	ns
Somata surface	452.5 ± 17.82 v*s* 320.6 ± 11.66	*p* < 0.001	452.5 ± 17.82 vs 276.4 ± 14	*p* < 0.001	320.6 ± 11.66 vs 276.4 ± 14	ns
Total surface	2077 ± 132.1 vs 1267 ± 66.97	*p* < 0.001	2077 ± 132.1 vs 1171 ± 97.31	*p* < 0.001	1267 ± 66.97 vs 1171 ± 97.31	ns
Processes volume	779.2 ± 68.36 vs 451.5 ± 29.85	*p* < 0.001	779.2 ± 68.36 vs 319.2 ± 31.68	*p* < 0.001	451.5 ± 29.85 vs 319.2 ± 31.68	*p* < 0.01
Somata volume	299.1 ± 15.94 vs 182.2 ± 8.98	*p* < 0.001	299.1 ± 15.94 vs 124.2 ± 7.89	*p* < 0.001	182.2 ± 8.98 vs 124.2 ± 7.89	*p* < 0.001
Total volume	1078 ± 81.70 vs 633.7 ± 36.51	*p* < 0.001	1078 ± 81.70 vs 443.4 ± 36.61	*p* < 0.001	633.7 ± 36.51 vs 443.4 ± 36.61	*p* < 0.01

Values represent mean ± SEM and *P*-value. Astrocytic processes, somata and total surface mean and SEM are expressed as µm^2^, whilst astrocytic processes, somata and total volume mean and SEM are expressed as µm^3^. ns = non-significance

### Changes in double GFAP/GS-positive astrocytes morphology in SAD and FAD compared to ND human brain samples

**Fig. 3:**
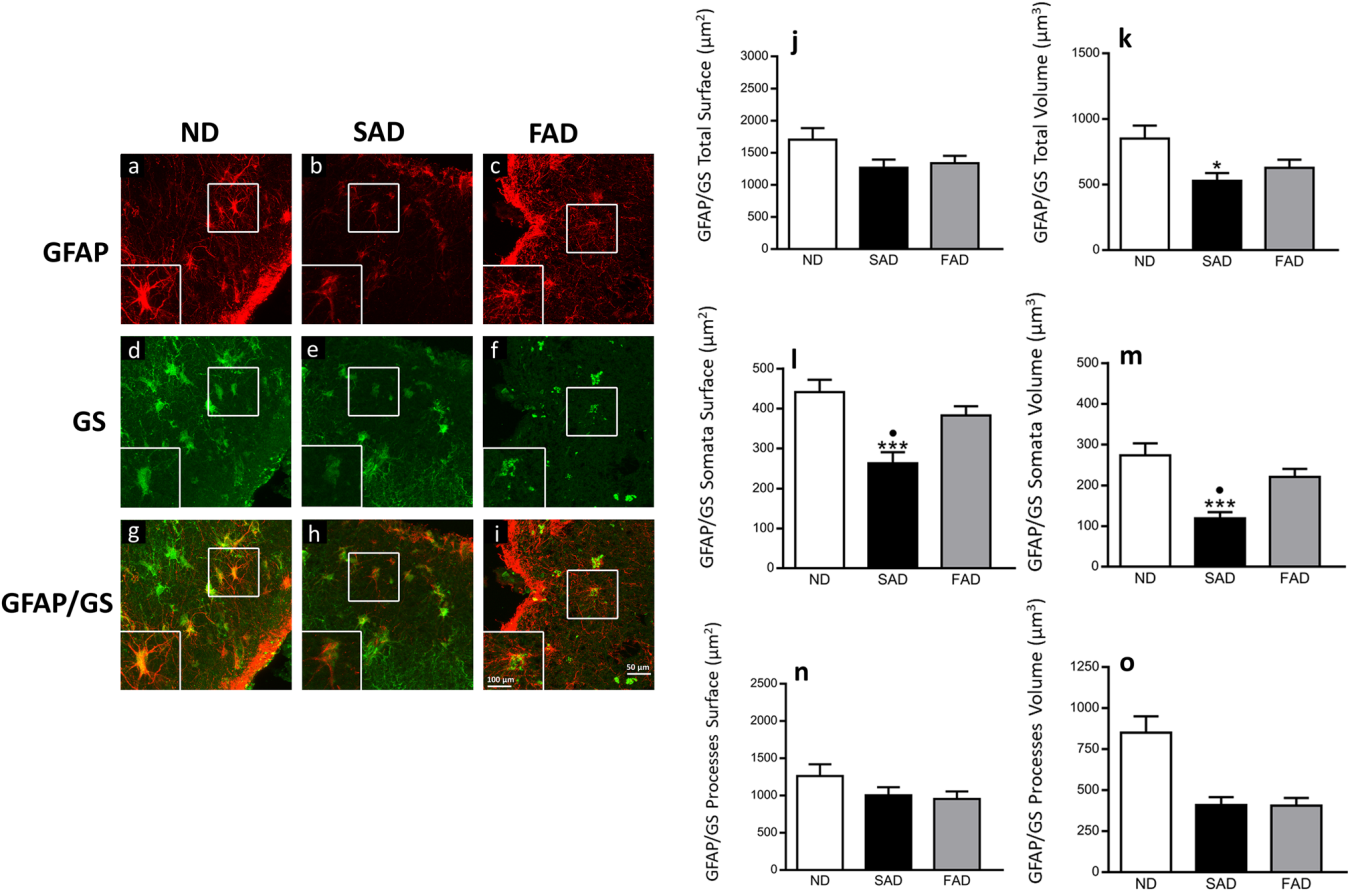
Dual confocal images (GFAP in red, GS in green and co-localisation of GFAP/GS in yellow) within the human entorhinal cortex of ND subjects (**a**, **d** and **g**), SAD (**b**, **e** and **h**) and FAD (**c**, **f** and **i**). Bar graphs showing the astrocytic total surface (**j**), total volume (**k**), somata surface (**l**), somata volume (**m**) processes surface (**n**) and processes volume (**o**). Bars represent means ± SEM (*p ≤ 0.05, ***p ≤ 0.001, comparing SAD or FAD with ND and ⋅*p* ≤ 0.05, comparing SAD with FAD)

Furthermore, we analysed the changes in the co-localised astrocytic populations of GFAP/ GS within the EC of the different AD subtypes and ND patients (Fig. 3, Table 3). When compared ND to SAD, we found no changes in the total GFAP/GS surface area (1704.0 ± 180.6 µm^2^ *vs.* 1267.0 ± 125.8 µm^2^; *p* > 0.05; Fig. 3j) nor when compared ND with FAD (1704.0 ± 180.6 µm^2^ *vs.* 1337.0 ± 117.1 µm^2^; Fig. 3j). On the other hand, the total volume of GFAP/GS-positive astrocytes was significantly reduced when comparing ND to SAD with a 37.88% decrease (850.1 ± 99.78 µm^3^ *vs.* 528.1 ± 59.23 µm^3^; *p* < 0.05; Fig. 3k) but non-significant changes were observed when comparing ND to FAD (850.1 ± 99.78 µm^3^ *vs.* 627.5 ± 61.18 µm^3^, *p* > 0.05; Fig. 3k) independently of the reduction. When compared the total volume of GFAP/GS, we observe a reduction in SAD when compared to ND (528.1 ± 59.23 µm^3^ *vs* 850.1 ± 99.78 µm^3^; *p* < 0.05; Fig. 3k). The somata surface of GFAP/GS-positive astrocytes showed a significant 40.35% decrease when comparing ND to SAD (441.1 ± 31.13 µm^2^ *vs.* 263.1 ± 27.29 µm^2^; *p* < 0.001; Fig. 3l) or non-significant differences when comparing ND to FAD (441.1 ± 31.13 µm^2^ *vs.* 382.7 ± 23.18 µm^2^; *p* > 0.05; Fig. 3l). On the other hand, somata volume showed a significant reduction of 56.41% when comparing ND to SAD (273.2 ± 29.49 µm^3^ *vs.* 119.1 ± 14.94 µm^3^; *p* < 0.001; Fig. 3m) and non-significant differences when comparing ND to FAD (273.2 ± 29.49 µm^3^ *vs.* 221.1 ± 19.63 µm^3^; *p* > 0.05; Fig. 3m). When compared the somata surface and volume of GFAP/GS, we observed a reduction in SAD when compared to FAD (263.1 ± 27.29 µm^2^ *vs* 382.7 ± 23.18 µm^2^; 119.1 ± 14.94 µm^3^ vs 221.1 ± 19.63 µm^3^; *p* < 0,05; Fig. 3l and m).

**Table 3:** Changes in double GFAP/GS-positive astrocytes morphological parameters in SAD and FAD compared to ND human brain samples

	ND vs SAD		ND vs FAD		SAD vs FAD	
Processes surface	1263.0 ± 156.4 vs 1004.0 ± 107.1	ns	1263.0 ± 156.4 vs 954.1 ± 100.7	ns	1004.0 ± 107.1 vs 954.1 ± 100.7	ns
Somata surface	441.1 ± 31.13 vs 263.1 ± 27.29	*p* < 0.001	441.1 ± 31.13 vs 382.7 ± 23.18	ns	263.1 ± 27.29 vs 382.7 ± 23.18	*p* < 0.05
Total surface	1704.0 ± 180.6 vs 1267.0 ± 125.8	ns	1704.0 ± 180.6 vs 1337.0 ± 117.1	ns	1267.0 ± 125.8 vs 1337.0 ± 117.1	ns
Processes volume	576.9 ± 75.59 vs 409.0 ± 48.15	ns	576.9 ± 75.59 vs 406.3 ± 45.71	ns	409.0 ± 48.15 vs 406.3 ± 45.71	ns
Somata volume	273.2 ± 29.49 vs 119.1 ± 14.94	*p* < 0.001	273.2 ± 29.49 vs 221.1 ± 19.63	ns	119.1 ± 14.94 vs 221.1 ± 19.63	*p* < 0.01
Total volume	850.1 ± 99.78 vs 528.1 ± 59.23	*p* < 0.05	850.1 ± 99.78 vs 627.5 ± 61.18	ns	528.1 ± 59.23 vs 627.5 ± 61.18	ns

Values represent mean ± SEM and *P*-value. Astrocytic processes, somata and total surface mean and SEM are expressed as µm^2^, whilst astrocytic processes, somata and total volume mean and SEM are expressed as µm^3^. ns = non-significance

GFAP/GS-positive astrocytic processes surface and volume demonstrated non-significant differences between ND, SAD and FAD samples. When compared ND to SAD, we found no statistical differences in the surface area (1263.0 ± 156.4 µm^2^ *vs.* 1004.0 ± 107.1 µm^2^; *p* > 0.05; Fig. 3n). Similarly, non-significant changes were observed in processes surface area when comparing ND to FAD (1263.0 ± 156.4 µm^2^ *vs.* 954.1 ± 100.7 µm^2^, *p* > 0.05; Fig. 3n). The volume of GFAP/GS-positive processes also showed non-significant differences. When compared ND to SAD, we found non-significant changes in the total processes volume (576.9 ± 75.59 µm^3^ *vs.* 409.0 ± 48.15 µm^3^; *p* > 0.05; Fig. 3o). Similarly, non-significant differences in the processes volume were observed when comparing ND with FAD samples (576.9 ± 75.59 µm^3^ *vs.* 406.3 ± 45.71 µm^3^; *p* > 0.05; Fig. 3o).

**Fig. 4:**
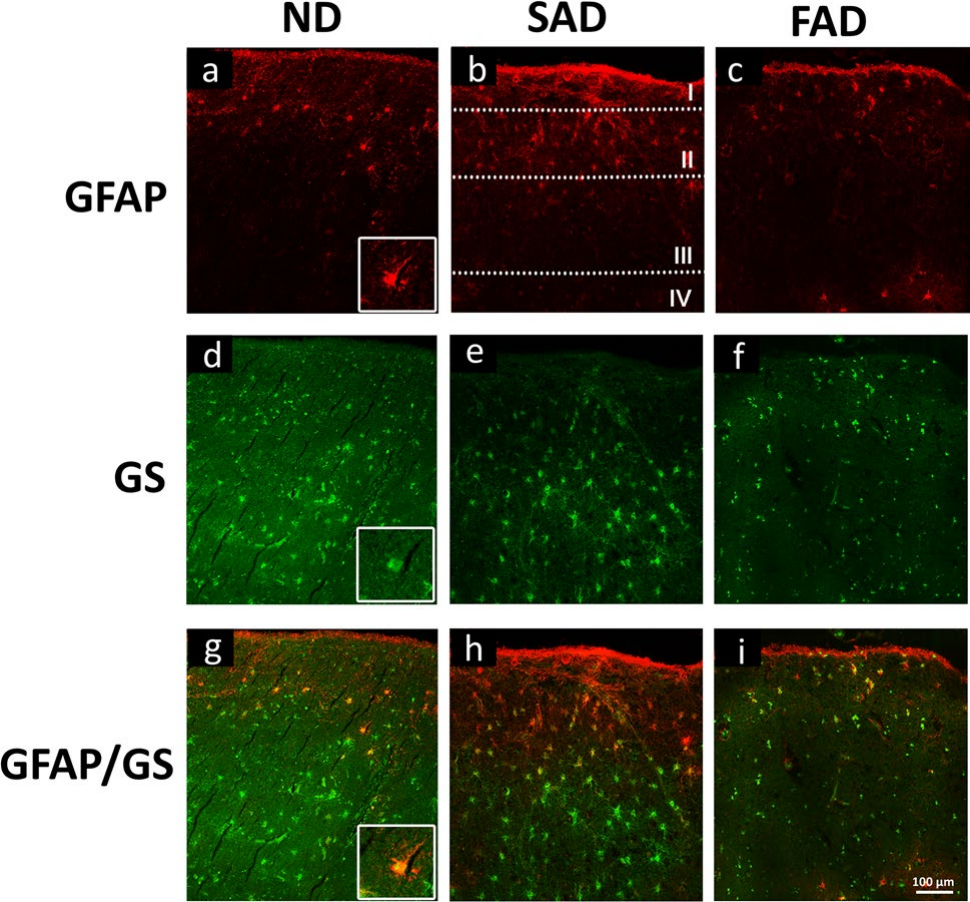
Confocal micrographs showing the different distribution of astrocytes labelled with GFAP (**a**, **b**, **c**), GS (**d**, **e**, **f**) and the co-localising cells (**g**, **h**, **i**) in the EC of ND subjects, SAD, FAD patient samples

After performing and in-depth analysis and determination of the numerical density, it was evident that astrocytic population was equivalent in the different layers of the EC and did not show percentage changes of all three populations GFA, GS and dual-labelled GFAP/GS neither in ND nor in SAD and FAD (Fig. 4).

## Discussion

In this study, we show a prominent atrophy of SAD and FAD astrocytes in the EC demonstrated by the decrease in size and volume of GFAP- and GS-positive astrocytes and in co-expressing GFAP/GS astrocytes.

In AD, the EC is the first affected region before it spreads to other areas and is essential for memory and learning (Bevilaqua et al. 2008). Studies in animals have tried to elucidate the astrocyte modifications related with normal ageing and AD, since astrocytes are key for brain physiology and pathology. Studies in 3xTg-AD revealed morphological changes in astrocytes associated with age-dependent cognitive decline and AD pathogenesis showing both gliosis and atrophy in the HPC (Olabarría et al. 2011), whereas in EC and PFC, gliosis is minimal or inexistent (Kulijewicz-Nawrot et al. 2013). Moreover, astroglia atrophy is also accompanied by a glutamate homeostatic alteration by the glutamine (Glu-Gln) which also in consequence affects the Glutamine-Glutamate-GABA metabolic cycle as well as the shuttle (Eid et al. 2012; Andersen et al. 2022) fundamental to sustain neurotransmission and neurotransmitter recycling directly linked to astrocyte energy metabolism. Astrocytes in AD suffer important metabolic changes as we have clearly shown GS-immunoreactive astrocytes reduction in the HPC and PFC but not in the EC (Olabarría et al. 2011; Yeh et al. 2013; Kulijewicz-Nawrot et al. 2013). All these findings were found in the 3xTg-AD animal model (Olabarría et al. 2011; Yeh et al. 2013; Kulijewicz-Nawrot et al. 2013; Rodríguez et al. 2023). This atrophy of GS-immunoreactive astrocytes leads to glutamate and GABA maintenance impairing since both glutamate and GABA are generated via GS, compromising transmission in the EC circuitry and projections towards the HPC and PFC (Rodríguez et al. 2014). This is clearly evident with reduced activity, synaptic transmission and of course the regional homeostasis (Eid et al. 2012; Rodríguez et al. 2014, 2016; Andersen et al. 2022) and mitochondrial dysfunction (Albrecht et al. 2007). However, to our knowledge, it is unknown if the above described GFAP- and GS-positive atrophic alterations described in the 3xTg-AD are also present in human SAD or FAD. Thus, the purpose of this study was to show evidence of these rarely measured changes. We showed that the GFAP and GS atrophy appeared in all layers of the EC in either SAD and FAD but was never present in ND independent of the layer or age, on the contrary to what occurs in animal models (Yeh et al. 2013). GFAP atrophy appears as an important reduction of primary processes and a massive reduction of secondary and distal processes in the EC layers. This atrophy shown in both SAD and FAD in our knowledge is a novel observation. Since always has been considered the neuroinflammation processes associated with AD Pathology (Singh 2022). We consider that this has always been somehow biased since few in-depth studies have been performed till the present manuscript and even less comparing both SAD and FAD.

On the contrary to our observation, a variety of post-mortem studies have shown astrogliosis in the EC of AD human samples (Muramori et al. 1998; Porchet et al. 2003; Vanzani et al. 2005). Furthermore, microarray analysis also showed a decrease in gene transcription of astrocytic cytoskeleton proteins in AD, implying a down-regulation of astrocytic cytoskeleton (Simpson et al. 2011) consistent with atrophy. The spatiotemporal incidence of astroglia atrophy is consistent with the pathological hallmarks of AD. The changes in EC astrocytes are observed mainly within layers II, III, and IV at early onset; layer V was affected at middle onset, whilst at a very late onset, they are restricted layer VI as also described previously in patients by MRI in EC, neocortex and hippocampus (Pini et al. 2016). Nevertheless, it should be noted that there is neither recovery of atrophic astrocytes at any stage nor cell loss as indicated by the Nv (Fig. 4). As described previously in animal models, astroglial atrophy reduced synaptic coverage and decreased metabolic support to neurons (Heneka et al. 2010; Verkhratsky et al. 2010). EC, therefore, is important for cognitive functions and is homologous to the dorsolateral PFC in primates and humans (Ongür et al. 2000; Hoover et al. 2007; Milà-Alomà et al. 2020).

Astroglia releases trophic factors, sustains metabolic support, and extracellular ion buffering supporting neuronal connectivity (Rodríguez et al. 2023; Verkhratsky et al. 2010; Nedergaard et al. 2003; Lalo et al. 2011). Therefore, the SAD and FAD astroglial atrophy observed in EC may result in impaired synaptic connectivity and EC homeostasis (Verkhratsky et al. 2010). It is likely that astrocyte atrophy in EC might compromise its output to other areas, especially the HPC, resulting in loss of synapses and/or dendritic spines causing long-term potentiation (Oddo et al. 2003; Bertoni-Freddari et al. 2008; Noristani et al. 2011). We do not have to forget that this atrophy could be directly related with the oxidative stress via NADPH oxidase 4 (NOX4) impairing mitochondrial function (Park et al. 2021), affecting the morphology, functionality and metabolism, as well as glutamate and GABA production together with their dysregulation in neurodegenerative diseases (Verkhratsky and Nedergaard 2014; Milà-Alomà 2020; Sood et al. 2021). In addition, there is and evident morphological difference (size and shape) and functionality together with gene expression changes that, as mentioned above, could play a role in mitochondrial impairment (Park et al. 2021; Arranz et al. 2019). All the above points out the differences on GS affection in the EC of 3xTg-AD compared to the human EC suggesting different astrocyte functionality between the mice animal model EC and the human AD (Rodríguez et al. 2014; Arranz et al. 2019).

The severe atrophy observed in both SAD and FAD would also imply, in addition to astrocytic atrophy, an important Neurovascular Unit (NVU) alteration via the release of astrocyte extravascular vesicles (A-EVs) (González-Molina et al. 2021). In a recent study, SAD was modelled with human induced pluripotent stem cell (hiPSC)-derived organoids and treated with serum to replicate the blood brain breakdown in AD recapitulating Aβ and p-Tau accumulation and a reduced function of astrocytes (Chen et al. 2021). In a similar study, carried out with iPSC-derived astrocytes from FAD and SAD patients, astrocytes exhibited pathological and morphological changes (Jones et al. 2017) with no significant difference in the relative proportions of each cell type between the FAD and SAD astrocyte groups.

Surprisingly our study has demonstrated that despite the astrocyte atrophy, we do not have any reduction in the number of cells. In this regard, S100β is a trophic factor that marks the largest population of astrocytes (Ogata and Kosaka 2002; Araque et al. 1999) and occupy the entire extent of the EC (Rodríguez et al. 2023). S100β was overexpressed in brain tissue of patients with AD and Down syndrome (Griffin et al. 1989; Jørgensen et al. 1990; Sheng et al. 1994). All these findings are in agreement with our recent observations showing an increase in S100β expression in 3xTg-AD (Rodriguez et al. 2023) responding to the fact that there is not astrocytic death in the EC of AD patients, as shown in the present study.

Finally, the anatomical differences observed in this study amongst SAD and FAD can be explained by the fact that the onset of SAD is decades latter than the onset of FAD (Hunter et al. 2013), and therefore, the atrophy in FAD should be less prominent.

Considering all these new results in SAD and FAD, together with our previous results in the 3xTg-AD mouse model, we could state that AD is not only a neuropathological process, but also an early and chronic gliopathology affecting brain areas prior to neuronal alterations. Thus, therapeutic approaches targeting simultaneously glial and neural impairments might be of relevance for AD treatment.

## Data Availability

Enquiries about data availability should be directed to the corresponding author.
